# Quantitative left ventricular rotational mechanics in Duchenne and Becker muscular dystrophy patients

**DOI:** 10.1186/1532-429X-17-S1-Q34

**Published:** 2015-02-03

**Authors:** Zhe Wang, Meral Reyhan, Nancy Halnon, Sarah N Khan, Paul J Finn, Pierangelo Renella, Daniel B Ennis

**Affiliations:** 1 Bioengineering, University of California Los Angeles, Los Angeles, CA, USA; 2 Radiological Sciences, University of California Los Angeles, Los Angeles, CA, USA; 3 Biomedical Physics Interdepartmental Program, University of California Los Angeles, Los Angeles, CA, USA; 4 Pediatrics, University of California Los Angeles, Los Angeles, CA, USA; 5 Pediatric Cardiology, Children's Hospital of Orange County, Orange, CA, USA

## Background

Duchenne/Becker muscular dystrophy (D/BMD) is associated with early onset cardiomyopathy, with cardiac fibrosis amongst the earliest manifestations. Changes in conventional estimates of cardiac function (e.g. ejection fraction, EF) may occur late in Duchenne and Becker muscular dystrophy (DMD/BMD). Cardiac MRI tagging is a noninvasive imaging biomarker for quantifying ventricular dysfunction in DMD/BMD cardiomyopathy. In particular, estimates of ventricular rotational mechanics (e.g. peak twist or normalized untwisting rate) may provide insight to early ventricular dysfunction. Furthermore, myocardial fibrosis in DMD/BMD patients is frequently reported and could significantly impact LV rotational mechanics. However, the functional consequences of myocardial fibrosis in these patients are incompletely understood. The objective of this study was to quantify LV rotational mechanics in pediatric DMD/BMD patients with normal EF (N-EF) or low EF (L-EF) and with (f+) or without (f-) fibrosis.

## Methods

Seventeen (N=17) male pediatric subjects (13.7±4.5 years old) genetically diagnosed with DMD/BMD consented to participate in an IRB approved study. Each patient underwent a cardiac 3T MRI exam that included evaluation of functional status with cine MRI, cardiac tagging MRI, and ventricular scar evaluation. Ten (M=10) non-aged-matched (29±4.3 years old) healthy volunteers were also evaluated to provide context for interpreting the pediatric data. LV mass, LVESV, LVEDV, and LVEF were calculated. The normal EF cut-off was >55.9% for 8-15 year-olds (Danielle et. al. JMRI 2009, 29: :552-559) and >53.2% for 16-20 year-olds (Sarikouch et. al. Circulation 2010 Jan;3(1):65-76). The presence or absence of myocardial fibrosis was determined by consensus agreement. Comparisons were made using t-tests with Holm-Sidak correction.

## Results

The volunteers had LV peak twist (LV-PT) of 12.2 ± 2.6° and lower LV normalized untwist rate (LV-NUR) -12.5 ± 2.1° s^-1^. The table shows lower LV-PT and LV-NUR in the EF-n/f- group compared to controls. LV-PT and LV-NUR significantly decreased in the N-EF/f+ group. LV-PT and LV-NUR were further decreased in the L-EF/f+ and were lowest in the L-EF/f+ group. T-tests with Holm-Sidak correction showed significant difference between L-EF/f- and L-EF/f+ in LV-PT. No significant difference was detected for LV-NUR.

## Conclusions

LV-PT and LV-NUR are lower in patients with DMD/BMD despite normal EF, compared to the healthy volunteers. Both low EF and the presence of fibrosis are associated further decreased LV-PT and LV-NUR. Fibrosis alone has a significant effect on LV rotational mechanics and should be further evaluated.

## Funding

AHA 13BGIA14530010 to Daniel B. Ennis, AHA 11PRE6080005 to Meral L. Reyhan

**Table 1:** 

	Normal (EF-N)		Abnormal (EF-L)	
Fibrosis (-)	LV-PT: 9.3± 2.2°	N=6	LV-PT: 8.7± 1.4°	N=2
				
	LV-NUR: -11.1± 3.2° s-1		LV-NUR: -10.5± 1.2° s-1	

Fibrosis (+)	LV-PT: 7.8± 2.2°	N=6	LV-PT: 5.3 ±0.5°	N=3
				
	LV-NUR: -9.1± 2.2° s-1		LV-NUR: -8.3± 0.5° s-1	

